# The ubiquitin ligase FbxL7 regulates the Dachsous-Fat-Dachs system in *Drosophila*

**DOI:** 10.1242/dev.113498

**Published:** 2014-11

**Authors:** Mariana Rodrigues-Campos, Barry J. Thompson

**Affiliations:** 1 Cancer Research UK - London Research Institute, Lincoln's Inn Fields, London WC2A 3LY, UK; 2 GABBA, ICBAS, Universidade do Porto, 4050-313 Porto, Portugal

**Keywords:** Dachsous, *Drosophila*, Fat, Hippo, Planar polarity

## Abstract

The atypical cadherins Dachsous (Ds) and Fat (Ft) are required to control the size and shape of tissues and organs in animals. In *Drosophila*, a key effector of Ds and Ft is the atypical myosin Dachs, which becomes planar polarised along the proximal-distal axis in developing epithelia to regulate tissue size via the Hippo pathway and tissue shape via modulating tension at junctions. How Ds and Ft control Dachs polarisation remains unclear. Here, we identify a ubiquitin ligase, FbxL7, as a novel component of the Ds-Ft-Dachs system that is required to control the level and localisation of Dachs. Loss of FbxL7 results in accumulation of Dachs, similar to loss of Ft. Overexpression of FbxL7 causes downregulation of Dachs, similar to overexpression of the Ft intracellular domain. In addition to regulating Dachs, FbxL7 also influences Ds in a similar manner. GFP-tagged FbxL7 localises to the plasma membrane in a Ft-dependent manner and is planar polarised. We propose that Ft recruits FbxL7 to the proximal side of the cell to help restrict Ds and Dachs to the distal side of the cell.

## INTRODUCTION

How animal cells cooperate to build tissues of particular forms remains a fundamental unsolved problem in biology. One molecular system that controls tissue size and shape in animals is the Dachsous (Ds)-Fat (Ft) cadherin system (reviewed by Goodrich and Strutt, 2011; Lawrence et al., 2008; Reddy and Irvine, 2008). Ds and Ft encode large atypical cadherins that interact heterotypically to form cell-cell junctions in epithelia and are required to control tissue form in both *Drosophila* and mice (Aigouy et al., 2010; Baena-Lopez et al., 2005; Bosveld et al., 2012; Bryant et al., 1988; Clark et al., 1995; Garoia et al., 2000; Mao et al., 2011a,b; Matakatsu and Blair, 2004; Saburi et al., 2008). The Ds-Ft system is known to induce a molecular polarity in the plane of the epithelium, and this planar polarity has at least three distinct consequences, including control of tissue growth via regulation of the Hippo signalling pathway (Cho et al., 2006; Pan et al., 2013; Rogulja et al., 2008; Willecke et al., 2008), control of tissue morphogenesis by modulating tension at cell-cell junctions (Baena-Lopez et al., 2005; Bosveld et al., 2012; Mao et al., 2011b), and control of the orientation of hairs, bristles and eye ommatidia in *Drosophila*, in part by modulating the Frizzled system of planar cell polarity (Adler et al., 1998; Ayukawa et al., 2014; Casal et al., 2006; Yang et al., 2002).

One important effector of Ds and Ft is the atypical myosin Dachs, which is thought to bind to the Ds intracellular domain and becomes planar polarised towards the distal side of each cell in the developing *Drosophila* wing or eye epithelium (Brittle et al., 2012; Mao et al., 2006, 2011b; Rogulja et al., 2008). Ds and Ft can also themselves become planar polarised, which may contribute to the polarisation of Dachs itself (Ambegaonkar et al., 2012; Bosveld et al., 2012; Brittle et al., 2012). Dachs then generates tension at distal cell-cell junctions to orient cell shapes, cell divisions or cell-cell re-arrangements to drive tissue elongation along the proximal-distal axis of various fly epithelia (Bosveld et al., 2012; Mao et al., 2011b). In addition, Dachs can signal to the nucleus via the Hippo pathway effector Yki (YAP/TAZ in mammals) to promote cell proliferation and tissue growth (Cho et al., 2006; Mao et al., 2006; Rogulja et al., 2008). Notably, Dachs appears to be dispensable for planar polarisation of the Frizzled system, and the ability of Ds and Ft to polarise hairs and bristles, a process that may instead depend on microtubules (Harumoto et al., 2010; Mao et al., 2006). Here, we focus on the Dachs-dependent roles of Ds and Ft in controlling tissue size and shape in *Drosophila*.

The global cues that orient Dachs polarisation along the proximal-distal axis are known: Dachs localises distally in response to graded expression of the Ds cadherin and also of Four-jointed (Fj), a kinase that modulates Ds-Ft interactions (Bosveld et al., 2012; Brittle et al., 2012, 2010; Ishikawa et al., 2008; Mao et al., 2006, 2011b; Rogulja et al., 2008; Simon et al., 2010). The gradients of Ds and Fj are opposing, such that Ds is highly expressed at the proximal end of the tissue and Fj is highly expressed at the distal end of the tissue. Yet, how epithelial cells read the slope of these gradients and translate this information into a planar polarised localisation of Dachs is still unknown. Here, we identify the ubiquitin ligase FbxL7 as a novel component of the Ds-Ft system that is crucial to control Dachs levels and localisation at apical cell-cell junctions.

## RESULTS AND DISCUSSION

**Fig. 1.**
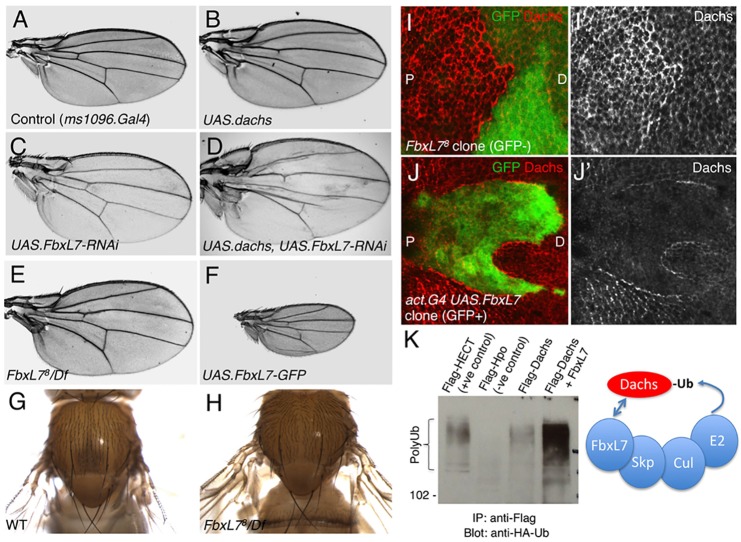
**The *FbxL7* gene encodes a ubiquitin ligase that targets Dachs for degradation.** (A) A *w MS1096.G4* control adult *Drosophila* wing. All wings are shown at the same magnification. (B) The *w MS1096.G4; UAS.dachs* wing is overgrown and slightly rounded in shape. (C) The *w MS1096.G4; UAS.FbxL7-IR (KK)* wing is overgrown and slightly rounded in shape. (D) *w* *MS1096.G4; UAS.FbxL7-IR/ UAS.dachs* wings have a dramatically enhanced size and rounded shape. (E) *w; FbxL7^8^/Df(3R)Exel7327* mutant wing showing overgrowth and rounded shape, similar to the RNAi phenotype. (F) Expression of FbxL7 with a UAS line (*w MS1096.G4; UAS.FbxL7GFP*) causes a *dachs*-like small and rounded wing phenotype. (G) Wild-type thorax (*w*). (H) *FbxL7* mutant thorax (*w; FbxL7^8^/Df(3R)Exel7327*) showing rounded shape and mild overgrowth. (I,I′) Clones of *FbxL7^8^* mutant cells (lacking GFP; *w hsflp; FRT82B GFP/FbxL7^8^ FRT82B*) upregulate the Dachs myosin cell-autonomously in the third instar wing imaginal disc. (J,J′) Clones of FbxL7-overexpressing cells (GFP positive; *w hsflp; act>CD2>G4 UAS.GFP /UAS.FbxL7GFP*) downregulate the Dachs myosin cell-autonomously in the third instar wing imaginal disc. (K) Expression of FbxL7 causes a dramatic increase in ubiquitylation of Flag-tagged Dachs in cultured *Drosophila* S2 cells. Model for FbxL7 triggering ubiquitylation of Dachs is shown on the right. Lanes were treated as follows: lane 1, HECT-FLAG 400 ng+ubi-HA 300 ng; lane 2, Hippo-FLAG 400 ng+ubi-HA 300 ng; lane 3, Dachs-FLAG 400 ng+ubi-HA 300 ng; lane 4, Dachs-FLAG 400 ng+ubi-HA 300 ng+FbxL7-MYC 300 ng.

We sought to identify novel components of the Ds-Ft system of planar polarity using a genetic approach. Using the *Drosophila* wing as a model system, we performed a transgenic RNAi screen with the VDRC library of UAS.inverted repeat hairpin RNAi lines. Overexpression of Dachs in the wing with the MS1096.Gal4 driver is known to produce an overgrown tissue that is also slightly rounded (Fig. 1A,B). We identified the novel gene *FbxL7* (*CG4221*) as producing a similar phenotype when silenced with RNAi (Fig. 1C). When combined with Dachs overexpression, RNAi of FbxL7 produces a strongly enhanced phenotype, producing a very large and rounded wing (Fig. 1D). To confirm the *FbxL7* RNAi phenotype, we generated *FbxL7* mutant alleles by P-element excision. We find that *FbxL7^8^* mutants phenocopy the RNAi lines, exhibiting enlarged and rounded wings (Fig. 1E). By contrast, overexpression of GFP-tagged FbxL7 in the entire wing resulted in small wings highly similar to *dachs* mutants (Fig. 1F) (Mao et al., 2006, 2011b). Notably, mutants in *FbxL7* also affected other tissues, such as the thorax, which was abnormally shaped and slightly overgrown, similar to hypomorphic alleles of *ft* (Fig. 1G,H) (Mao et al., 2006, 2011b). These phenotypes suggest that the *FbxL7* gene acts antagonistically to Dachs and is therefore likely to be a novel component of the Ft-Ds-Dachs system.

The *FbxL7* gene encodes an E3 ubiquitin ligase of the F-box leucine-rich repeat (LRR) family and is conserved from *Drosophila* to mammals. E3 ubiquitin ligases of this family recognise substrates via their LRR domains and recruit the Skp-Cullin-E2 module via their F-box domains to trigger ubiquitylation of the substrate and subsequent degradation by the proteasome. To examine the function of the *FbxL7* gene further, we stained for Dachs in *FbxL7^8^* mutant clones in the developing wing. We find that Dachs levels are upregulated in *FbxL7* mutant clones (Fig. 1I). Conversely, Dachs is downregulated in FbxL7-overexpressing clones (Fig. 1J). In addition, we performed ubiquitylation assays with HA-tagged ibiquitin in S2 cells, which demonstrate that ubiquitylation of Flag-tagged Dachs is strongly stimulated by co-expression with FbxL7 (Fig. 1K). Together, these results suggest that FbxL7 can ubiquitylate Dachs *in vitro* and promote removal of Dachs from the apical domain *in vivo*.

**Fig. 2.**
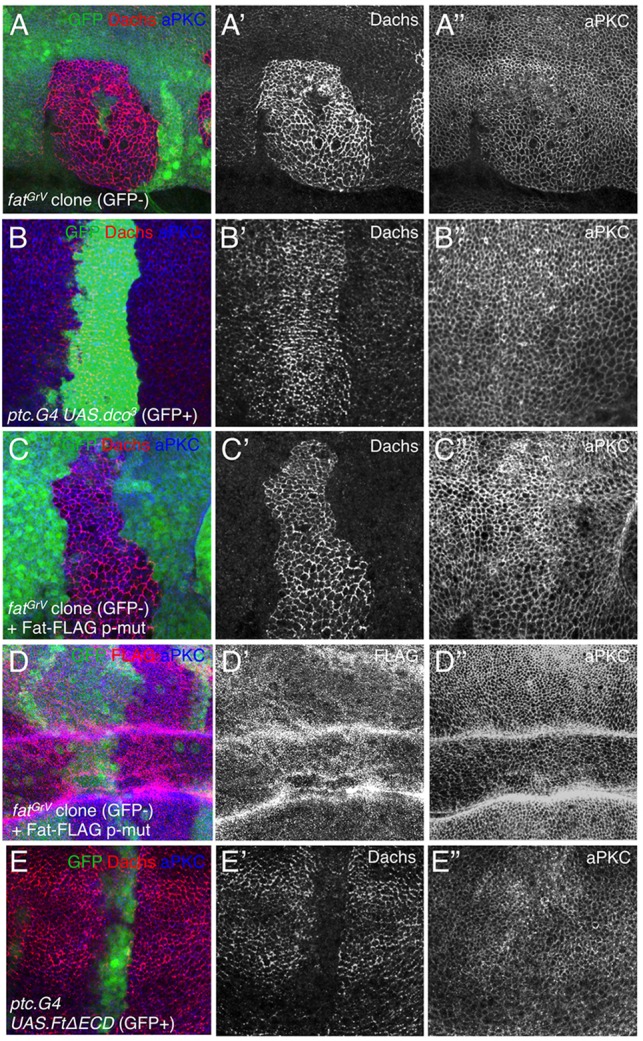
**Phosphorylation of the Fat intracellular domain by the Dco kinase promotes Dachs degradation in the wing imaginal disc.** (A-A″) *fat* mutant clones (GFP negative; *w hsflp; FRT40A fat^GrV^/ FRT40A GFP*) accumulate Dachs. (B-B″) Expression of dominant-negative Dco (GFP positive; *w; ptc.G4 UAS.GFP/UAS.dco^3^*) causes accumulation of Dachs. (C-C″) Rescue of *fat* mutant clones (GFP negative; *w hsflp; FRT40A fat^GrV^/ FRT40A GFP; {P[acman]-V5:Ft:FLAG^mV^}/+*) by expression of phopho-mutant Fat still produces accumulation of Dachs. (D-D″) Control experiment showing expression of Flag-tagged phospho-mutant Fat in *fat* clones (GFP negative; *w hsflp; FRT40A fat^GrV^/ FRT40A GFP; {P[acman]-V5:Ft:FLAG^mV^}/+*). (E-E″) Overexpression of Ft intracellular domain causes degradation of Dachs in *w; ptc.G4 UAS.GFP/+ ; UAS.ft^ΔECD^*/+.

Previous work has shown that loss of Ft also produces accumulation of Dachs, similar to loss of FbxL7 (Brittle et al., 2012) (Fig. 2A). This phenotypic similarity suggests that Ft and FbxL7 may function together to regulate Dachs. Ubiquitin ligases of the F-box family tend to bind to phosphorylated target proteins, and Ft is known to be phosphorylated on its intracellular domain by the Casein Kinase 1 ε protein Discs-overgrown (Dco) (Feng and Irvine, 2009; Sopko et al., 2009). We therefore wondered whether phosphorylation of Ft by Dco was also important for regulating Dachs levels. We induced clones overexpressing dominant-negative Dco (*UAS.dco^3^*) and find that Dachs levels are elevated within these clones, similar to *ft* mutant clones or *FbxL7* mutant clones (Fig. 2B). Furthermore, when *ft* mutant clones are rescued by expression of a *ft* transgene lacking the Dco phosphorylation sites [Ft p-mut (*ft*^mV^); Pan et al., 2013], Dachs levels are also elevated (Fig. 2C,D). By contrast, overexpression of the Ft intracellular domain (FtΔECD) resulted in degradation of Dachs (Fig. 2E). These findings suggest that the Ft intracellular domain, Dco kinase and FbxL7 act together to regulate Dachs *in vivo*.

**Fig. 3.**
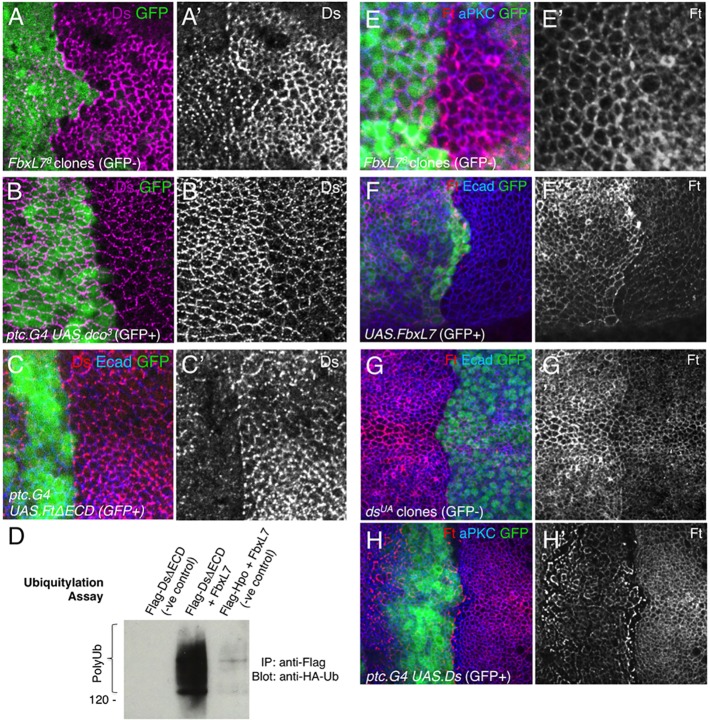
**FbxL7 promotes degradation of Dachsous and stabilisation of Ft in the wing imaginal disc.** (A,A′) Clones of *FbxL7* mutant cells (lacking GFP; *w hsflp; FRT82B GFP/FbxL7^8^ FRT82B*) upregulate Ds levels. (B,B′) Expression of dominant-negative Dco (GFP positive; *w; ptc.G4 UAS.GFP/UAS.dco^3^*) causes upregulation of Ds levels. (C,C′) Expression of Ft intracellular domain (GFP positive; *w; ptc.G4 UAS.GFP/+ ; UAS.ft^ΔECD^/+*) causes downregulation of Ds levels. (D) Expression of FbxL7 in S2 cells triggers ubiquitylation of DsΔECD. Lane 1, FLAG-ΔsDECD 400 ng+ubi-HA 300 ng; lane 2, FLAG-ΔsDECD 400 ng+ubi-HA 300 ng+FbxL7-MYC 300 ng; lane 3, Hippo-FLAG 400 ng+ubi-HA 300 ng+FbxL7-MYC 300 ng. (E,E′) *FbxL7* mutant clones (lacking GFP; *w hsflp; FRT82B GFP/FbxL7^8^ FRT82B*) mildly reduce Ft levels at the membrane (Ft appears more cytoplasmic) but increase Ft levels at the clone boundary. (F,F′) Expression of FbxL7 (GFP positive; *w; act>CD2>G4 UAS.GFP /UAS.FbxL7GFP*) causes upregulation of Ft levels at the membrane. (G,G′) Clones of *ds* mutant cells (lacking GFP; *w hsflp; FRT42D ds^UA^/FRT42D GFP*) upregulate Ft levels. (H,H′) Expression of Ds (GFP positive; *w; ptc.G4/+ ; UAS.ds^L14^/+*) causes downregulation of Ft levels, except at the clone boundary, where Ft is stabilised.

As Ds is known to become planar polarised to the distal side of the cell in a manner similar to Dachs (Brittle et al., 2012), we tested whether FbxL7, Dco and Ft might be involved in regulating Ds levels and localisation. In *FbxL7^8^* mutant clones, Ds levels are elevated and appear less well polarised in the wing disc (Fig. 3A). Ds is similarly elevated upon expression of dominant-negative Dco^3^ (Fig. 3B). By contrast, overexpression of Ft intracellular domain with the *ptc.Gal4* driver causes downregulation of Ds in the wing disc (Fig. 3C). In cultured S2 cells, overexpression of FbxL7 is able to ubiquitylate the Ds intracellular domain directly (Fig. 3D). These findings suggest a model in which Ft intracellular domain, Dco and FbxL7 act together to regulate both Ds and Dachs, possibly via directly ubiquitylating both proteins. Notably, as Dachs is a cytoplasmic protein, its ubiquitylation is expected to lead to proteasomal degradation. By contrast, Ds is a transmembrane protein and its ubiquitylation is expected to lead to endocytosis and lysosomal degradation. It is also plausible that endocytic removal of the entire Ds-Dachs complex may be promoted by FbxL7.

We next tested whether FbxL7 might also influence Ft itself. We find that *FbxL7^8^* mutant clones have only a mild effect on Ft, causing a slight decrease in Ft levels at the plasma membrane and an accumulation of Ft at the clonal boundary (Fig. 3E). More strikingly, overexpression of FbxL7 causes a clear increase in Ft levels (Fig. 3F). This finding might suggest that FbxL7 directly promotes Ft stabilisation at the membrane, yet could also be an indirect effect of FbxL7 via its regulation of Ds. We note that a clear increase in Ft levels is evident in mutant clones for *ds*, suggesting that turnover of Ds may account for the stabilisation of Ft induced by FbxL7 overexpression (Fig. 3G; see also Mao et al., 2009). Furthermore, increased Ds levels may account for the alteration of Ft in *FbxL7^8^* clones, because overexpression of Ds decreases Ft levels except at the clone boundary where Ft levels are increased (Fig. 3H).

**Fig. 4.**
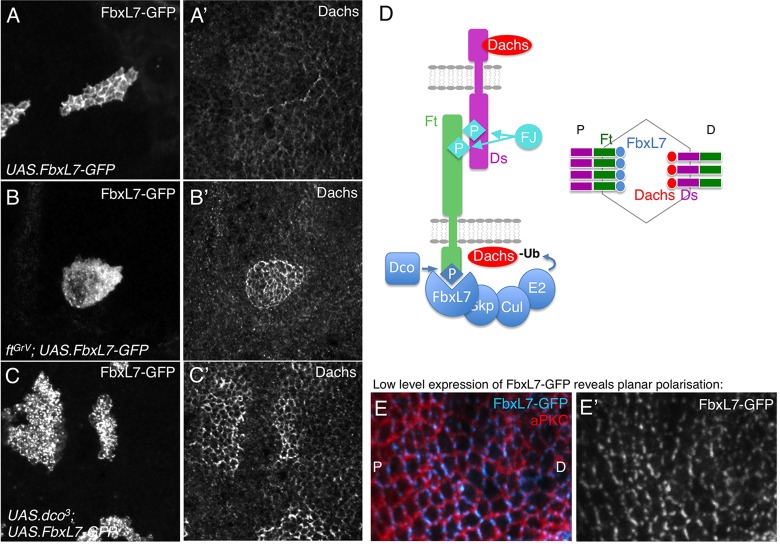
**Fat intracellular domain and Dco recruit and activate FbxL7 to degrade Dachs in the wing imaginal disc.** (A,A′) In *yw hsflp; FRT40A/ FRT40A, tubgal80; UAS.FbxL7GFP/tubgal4,* GFP-tagged FbxL7 localises to cell-cell junctions and downregulates Dachs when expressed in a clone of cells in the wing imaginal disc. (B,B′) In *yw hsflp; FRT40A/ FRT40Afat^GrV^, tubgal80; FbxL7GFP/tubgal4*, GFP-tagged FbxL7 fails to localise to cell-cell junctions in *ft* mutant clones and instead has a more cytoplasmic and punctate localisation. (C,C′) In *yw hsflp; FRT40A/ FRT40A, tubgal80; FbxL7GFP, UAS.dco^3^/tubgal4*, GFP-tagged FbxL7 fails to localise to cell-cell junctions when co-expressed with dominant-negative Dco and instead has a more cytoplasmic and punctate localisation. (D) Model for FbxL7 recruitment to Ft and ubiquitylation of Dachs to promote planar polarisation. (E,E′) Low-level expression of FbxL7-GFP with the MS1096.Gal4 driver in *w MS1096; UAS.FbxL7GFP/+* at 18°C reveals planar polarisation, whereas aPKC localises around the entire apical cell-cell junction.

The above results suggest a close relationship between the function of the Ft intracellular domain, Dco and FbxL7. We therefore tested whether phosphorylated Ft intracellular domain might recruit FbxL7 to the plasma membrane. We examined the localisation of GFP-tagged FbxL7 expressed in clones and find that FbxL7-GFP localises to apical cell-cell junctions (Fig. 4A). By contrast, when FbxL7-GFP is expressed in *ft* mutant clones, it localises to the cytoplasm in a punctate pattern (Fig. 4B). A similar punctate pattern is observed when FbxL7-GFP is co-expressed with dominant-negative Dco^3^ (Fig. 4C). Notably, the loss of Dachs that is normally induced by expression of FbxL7-GFP fails to occur when it is not recruited to the membrane by Ft and Dco (Fig. 4A-C). These findings support the notion that phosphorylated Ft recruits FbxL7 in order to downregulate Dachs (Fig. 4D). This model predicts that FbxL7 itself should be planar polarised to the proximal side of cells, where Ft is thought to be most concentrated and active, whereas Dachs localises to the distal side of cells away from FbxL7 and in a complex with Ds (Bosveld et al., 2012; Brittle et al., 2012). Accordingly, low-level expression of FbxL7-GFP with *ms1096.G4* reveals a planar polarised localisation, presumably to the proximal side of wing epithelial cells where Ft is known to concentrate (Brittle et al., 2012) (Fig. 4E).

The above findings identify the FbxL7 ubiquitin ligase as a novel component of the Ds-Ft-Dachs system. FbxL7 is recruited to the membrane by Ft, where it then acts together with Ft and the Dco kinase to promote degradation or removal of both Dachs and Ds. The effect of FbxL7 loss and gain of function on Dachs levels are particularly strong and the phenotypic consequences in adult *Drosophila* closely resemble gain and loss of Dachs function, respectively. Our *in vitro* ubiquitylation assays suggest that FbxL7 can directly ubiquitylate Dachs, which is predicted to lead to its proteolytic degradation. In addition, we observe that FbxL7 can also ubiquitylate the Ds intracellular domain *in vitro* and can modulate the level and localisation of Ds *in vivo*. It remains possible that FbxL7 acts indirectly by stabilising or activating Ft, which then acts via a different mechanism to degrade or remove Ds and Dachs proximally. We favour the direct model because of its simplicity and because ubiquitylation is generally thought to promote degradation, rather than stabilization, of proteins.

These observations suggest a model in which Ft, which has been reported to localise proximally (Brittle et al., 2012), recruits FbxL7 to the proximal side of the cell to help restrict Dachs and Ds to the distal side of the cell. Our results also suggest that polarised Ds may also promote degradation or removal of Ft on the distal side so that Ft concentrates proximally, thereby assisting polar Ds-Ft bridge formation. Thus, there appears to be mutual antagonism between Ds and Ft within the same cell, as well as heterotypic Ds-Ft bridge formation between neighbouring cells, an event that then leads to loss of Dachs proximally and recruitment of Dachs distally. Such a mechanism might explain how this system can become planar polarized; however, it is still unclear how the system is able to read the slope of the Ds and Fj gradients continuously, rather than switch to a more permanently polarised state.

Notably, the degree of Dachs polarisation – and the strength of its effect on Hippo signalling and tissue growth – correlates with the steepness of the Ds and Fj gradients, indicating that cells can obtain both vectorial information and a measure of steepness at the same time from the Ds-Ft system (Brittle et al., 2012; Casal et al., 2006; Mao et al., 2011b; Rogulja et al., 2008; Willecke et al., 2008). These features of the Ds-Ft system match very well with those proposed for the hypothetical gradients originally conceived following surgical manipulation of insect development and regeneration (Lawrence, 1966, 1970; Lawrence et al., 2008; Stumpf, 1966). Our identification of FbxL7 as a key player in this system will help enable further work to understand how the system can translate the steepness of the gradient into the degree of Dachs polarisation.

## MATERIALS AND METHODS

### *Drosophila* genotypes and immunostaining

All fly strains are described in FlyBase, with the exception of the FbxL7^8^ and UAS.FbxL7GFP transgenic fly lines, which were generated for this study. FbxL7^8^ is a small ∼1.44 kb deletion of the 5′ coding region and 1st intron of FbxL7, and is a pupal-lethal allele (viable escapers over a deficiency). Mosaic tissues were generated using the FLP/FRT, MARCM and Act>FlpOUT system with a heat-shock promoter (hs) to drive the expression of the FLP recombinase. Clones were induced by heat shocking larvae at 60 h (±12 h) of development; larvae were dissected at the third instar stage. Third instar wing imaginal discs were fixed in 4% paraformaldehyde in PBS followed by permeabilization and blocking in PBS with 1% BSA and 0.2% Triton X-100. Primary antibodies used for staining include rat anti-Fat (David Strutt, The University of Sheffield, UK; 1:250), rabbit anti-Dachsous (David Strutt; 1:100), rat anti-Dachs (David Strutt, 1:500), rabbit anti-aPKC (Santa Cruz; 1:500; sc-216), rat anti-Ecad (DSHB; 1:100), rabbit anti-GFP (AMS Biotechnology; 1:250; TP401), mouse anti-GFP (1:250; Roche; 11814460001) and mouse anti-V5 (1:100; Abcam; 27671). Fluorescent stains were captured on a Zeiss Upright 710 confocal microscope.

### FbxL7 expression constructs

cDNA fragments encoding FbxL7 (CG4221) were cloned into the *Drosophila* Gateway expression vector pAWM for expression of the peptide fused to six MYC affinity tag at the C terminus. *Drosophila* S2 cells were transfected with Effectene transfection reagent (QIAGEN) and grown in *Drosophila* Schneider medium (Invitrogen) containing 10% FBS, penicillin and streptomycin. The same construct was cloned into the *Drosophila* Gateway expression vector pPWG and injected into flies to generate UAS.FbxL7-GFP line (Bestgene).

### Immunoprecipitation and immunoblot analysis

Immunopecipitation of FLAG-Ds, Dachs-FLAG, Hippo-FLAG or HECT-FLAG with Ubi-HA from transfected *Drosophila* S2 cells was performed using the anti-FLAG M2 affinity gel (Sigma) standard method. In detail, S2 cells were seeded at ∼3.0×10^6^ cells/well in a 6-well plate and allowed 20 min to attach. Effectene was used for transfection according to manufacturer's protocol.

Three days post-transfection samples were treated with MG132 5 μM (Sigma) and calpain inhibitor I 5 μM (Sigma) for 4 h. Cells were harvested and lysed in 1% Triton X-100 standard buffer (300 μl/well). As ‘input’, 40 μl lysate +25 μl 4× loading buffer was stored at −20°C. For the immunoprecipitation (IP), 500 μg total protein+mouse anti-Flag-agarose beads were used. Lysates+complete lysis buffer were added to beads and rolled gently for 2 h at 4°C. Beads were centrifuged and supernatant discarded. Beads were resuspended in 0.5 ml complete lysis buffer and rolled at 4°C for 5 min (this wash was repeated three times). Supernatant was removed from beads, using gel-loading tips. Reducing buffer and sample buffer were added and sample was boiled at 70°C for 10 min. Western blot was performed and transfer was carried out using iBlot Dry Transfer System (Invitrogen). The following antibodies were used in western blot analysis: rat anti-HA, 1:5000 (Roche, 11867423001), mouse anti-FLAG, 1:1000 (Sigma, F1804) and rabbit anti-MYC, 1:5000 (Santa Cruz, sc-40). Secondary antibodies were anti-rabbit peroxidase-conjugated antibody, 1:10,000 (Thermoscientific 31460), anti-mouse peroxidase-conjugated antibody, 1:10,000 (Thermoscientific, 31430) and anti-rat peroxidase-conjugated antibody, 1:10,000 (Thermoscientific, 31470).
